# Postpartum Depression and Birth Experience in Russia

**DOI:** 10.11621/pir.2021.0103

**Published:** 2021-03-20

**Authors:** Vera A. Yakupova, Anna Suarez

**Affiliations:** a Lomonosov Moscow State University, Moscow, Russia; b University of Helsinki, Helsinki, Finland

**Keywords:** postpartum depression (PPD), birth satisfaction, maternal mental health, prevention of postpartum depression, doula support

## Abstract

**Background:**

In European countries, postpartum depression (PPD) occurs in 13–19% of women. The statistics indicate that postpartum depressive disorders affect up to 300,000 women in Russia annually. There is still an extremely acute lack of psychological comfort provided to women during labor in Russia.

**Objective:**

To our knowledge, ours is the first study that examines the association between childbirth experience and the risk of PPD in Russia.

**Design:**

We collected data from 190 Russian-speaking mothers, ages 19 to 46, (M = 32 ± 4.3) two months after their delivery.

**Results:**

Birth satisfaction and physical well-being two months after delivery were significantly inversely associated with PPD. Birth satisfaction negatively correlated with the perceived severity and unpredictability of labor, and positively correlated with physical well-being two months after delivery. The presence of a partner and a personal midwife or doula at birth was associated with higher birth satisfaction.

**Conclusions:**

Our results emphasize the significance of childbirth satisfaction in the context of PPD and suggest the importance of individual professional support during labor.

## Introduction

Postpartum depression (PPD) occurs in 13–19% of women globally (O'Hara & McCabe, 2013), with an estimated overall prevalence of 17% among healthy mothers without a prior history of depression (Shorey et al., 2018). However, there is a significant heterogeneity of the PPD prevalence across nations, ranging from 3% in Singapore to 38% in Chile (Hahn-Holbrook,Cornwell-Hinrichs, & Anaya, 2018). Therefore, it is important to study PPD within national contexts.

Higher rates of PPD have been observed in nations with significantly higher rates of income inequality, maternal and infant mortality, and women of childbearing age working more than 40 hours a week (Hahn-Holbrook, Cornwell-Hinrichs, & Anaya, 2018). In Russia, income inequality has increased significantlyover the past 30 years (Morgan & Neef, 2020), and while rates of both maternal and infant mortality have been improving, they are still considerably higher than in the European countries (Danishevski, Balabanova, Parkhurst, & McKee, 2003). While these factors indicate that we may expect high prevalence of PPD in Russia compared to the global trend, there is a substantial knowledge gap, since no comprehensive studies have addressed the incidence, prevalence, and risk factors for PPD in Russia.

An extensive World Health Organization (WHO) report in 2003 indicated a number of risk factors for PPD (Stewart et al.). The authors showed that a history of psychopathology before and during pregnancy, maternal neuroticism and difficult child temperament, lack of social support, and obstetric difficulties among others, present risk for development of PPD. While obstetric factors were reported to make a small but significant contribution to the development of PPD, the subjective experience of childbirth was not examined in this literature review.

Newer evidence, however, suggests that a traumatic childbirth experience plays a major role in the development of PPD (Bell & Andersson, 2016; Gosselin, Chabot, Béland, Goulet-Gervais, & Morin, 2016); our previous studies have corroborated this (Yakupova, 2018). In line with these studies, obstetric violence and medical interventions were associated with increased risk for PPD (Mohammad, Gamble, & Creedy, 2011; Silveira et al., 2019; Souza, Rattner, & Gubert, 2017; Xu Ding, Ma, Xin, & Zhang,2017). Obstetric violence includes being subjected to mistreatment, disrespect, abuse, negligence, and violation of human rights by health professionals (WHO, 2014), while medical interventions include emergency cesarean section, routine synthetic oxytocin administration, pre-labor rupture of membranes, and episiotomy.

In Russia, modern evidence- and ethics-based obstetric practices are still in their infancy, while the maternity healthcare system largely follows the conservative Soviet approach (Borozdina & Novkunskaya, 2020). The conservative approach includes a paternalistic style of communication, lack of ethical concern, outdated medical practices, and overall medicalization of birth, with medical personnel focusing primarily on the bureaucratic demands rather than on the patient’s needs and psychological comfort (Temkina, 2014).

Studies ndicate that maternal psychological well-being after childbirth is associated with a sense of security, the ability to make her own decisions, and continuous support during labor (Bohren et al., 2017; Saisto, Salmela, Nurmi, & Halmesmaki, 2001). However, in Russia, women frequently lack support during labor, since small regional maternity hospitals can still prevent partners from attending the birth, and a doula or private midwife is available only by paid contract, not through the public healthcare sector (Novkunskaya, 2017).

Taken all together, these factors indicate that women may be at higher risk for developing PPD in Russia. However, to our knowledge, no study has investigated this risk or protective factors against it in relation to PPD. Thus, the aim of our study was to examine the association between obstetric factors, childbirth satisfaction, and continuous support during labor with PPD symptoms two months after delivery.

We hypothesized that 1) a higher level of PPD symptoms was associated with a lower level of childbirth satisfaction, poorer physical well-being after delivery, and delivery mode (emergency cesarean vs. elective cesarean vs. vaginal birth); and 2) a higher level childbirth satisfaction was associated with delivery mode (vaginal vs. elective cesarean vs. emergency cesarean birth), better physical well-being after delivery, and mode of birth support (support by a partner, doula or private midwife vs no support).

## Materials and Methods

### Study design

Data collection took place from June 2018 to February 2019. The women received an invitation to take part in the study through thematic communities and training classes for moms-to-be and new parents. Questionnaires were sent to respondents via e-mail after their written consent to participate was received. The participants were interviewed two months after delivery (the average age of the children was 1.9 ± 0.22 months). The inclusion criteria were 1) being at least 18 years old, and 2) giving birth no more than two months ago.

### Participants

One hundred ninety (190) women participated in the study. All participants spoke Russian and lived in big cities (population over 500,000). The characteristics of the sample are presented in the Table 1. More than 96 percent (96.3%) of the participants gave birth in maternity hospitals and 3.7% gave birth at home. More than one third (34.3% = 65) of the participants showed clinically significant depressive symptoms (*Table 1*).

**Table 1:** Characteristics of the sample (N = 190)

		M/N	SD/%	Range
Age at testing (years)		32	4.3	19-46
Education	Upper secondary/College	16	8.4%	
	Tertiary/University	174	91.6%	
Family status	Married	158	83%	
	Cohabiting with a partner	27	14.5%	
	Single	5	2.5%	
Time passed after the childbirth (months)		1.9	0.22	1.7-2.1
Gestational age		39.4	1.6	37-42
Parity	Primiparous	82	43%	
	Multiparous	108	57%	
Delivery mode	Vaginal	139	73%	
	Emergency cesarean	23	12%	
	Elective cesarean	28	15%	
Mode of birth support	No support	69	36.1%	
	Partner	75	39.3%	
	Doula/Private midwife	15	8.2%	
	Partner + Doula/Private midwife	31	16.4%	
EPDS score		7.9	2.3	0-21
EPDS scores ≥ 10 (clinically significant PPD symptoms, yes)		65	34.3%	
Physical health in 2 months after deliver		4.2	0.8	2-5
Perceived severity of labor		2.5	1.1	1-5
Expectations about labor		2.9	1.2	1-5
BSS-RI score		7.3	2.2	0-10
Type of the birth support	No support	7	2.6	0-10
	Partner	8	1.9	0-10
	Partner + doula/midwife	9	1.7	0-10
	Doula/midwife	7	2.1	0-10

*Note. EPDS = Edinburgh Postnatal Depression Scale. BSS-RI = Birth Satisfaction Scale Revised Indicator.*

48.7% of the participants gave birth in Moscow and the Moscow Region; 14.4% in other regions of the Russian Federation; 18.2% in European cities; 9.6% in Israel; and 9.1% in former USSR countries.

### Procedures

The Russian version (Yakupova, 2018; Cronbach’s α = 0.84) of the Edinburgh Postnatal Depression Scale (EPDS) (Cox, Holden, & Sagovsky, 1987)was used to estimate PPD symptoms. It is a 10-item questionnaire scale rated on a 4-point Likert scale, ranging from 0 to 3, which indicates how the mother has felt during the previous week. A score of 10 and higher is considered to indicate symptoms of depression.

The participants were asked to measure the discrepancy between their expectations about labor and real experience (“How closely did your expectations of childbirth match reality?”) on a 5-point Likert scale (1 = "did not at all" to 5 = "matched completely"). We also asked the participants to assess the perceived severity of their birth experience (“How hard was the birth experience for you?”) on a 5-point Likert scale (1 = "easy" to 5 = "extremely hard").

We used the Russian version (Yakupova, 2019; Cronbach’s α = 0.70) of the Birth Satisfaction Scale Revised Indicator (BSS-RI), a short 6-item self-report questionnaire, to assess birth satisfaction (the subscales include the level of stress and anxiety, feeling of control, and medical staff support)(Martin, Martin, & Redshaw, 2017). A 3-point Likert scale was used for each question (range 0-2), with the higher scores representing greater birth satisfaction.

We also asked the participants to assess their health condition at the moment of the screening (two months after delivery) on a 5-point Likert scale (1 = "very poor" to 5 = "very good"). Finally, we collected data on the participants’ socio-demographic characteristics, such as the number of children, the mode of delivery, gestational age, place and time of delivery, and type of delivery support.

### Statistical analysis

The main variables of the study were: PPD (based on EPDS scores); physical well-being after delivery; birth satisfaction (based on BSSR-RI scores); and the subjective severity and unpredictability of labor.

Spearman’s correlation coefficient was used to estimate the relationship between PPD, birth satisfaction, physical well-being, and the predictability and subjective severity of labor.

Multiple linear regression analysis examined the factors predicting PPD, where PPD was a dependent variable, and birth satisfaction, physical well-being after delivery, perceived severity of birth experience, and predictability of birth were entered in the model as the independent variables. The data met the assumptions for multiple regression analyses; the residuals were normally distributed after the square root transformation of the dependent variable. Model 1 explored unadjusted associations; Model 2 was adjusted for maternal age, gestational age at birth, and the number of children.

We used the Kruskal-Wallis test to assess the differences in the levels of PPD, physical well-being, and childbirth satisfaction between the groups with different kind of labor support (alone; with partner; with doula or midwife and partner; or with doula or midwife without partner) and mode of delivery.We analyzed the differences in individual support depending on the country of birth by the chi-square test.

All analyses were performed using IBM SPSS Statistics 22.

## Results

The level of childbirth satisfaction correlated negatively with PPD (rho = -0.28; p <0.01) (*Table 2*). Women with higher PPD scores more often perceived their labor as severe (rho = 0.21; p <0.01). A serious discrepancy between the reality and expectations of childbirth was negatively associated with satisfaction with childbirth (rho = -0.40; p<0.01).

**Table 2:** Levels of pre- and postnatal depression birth experience and physical well-being

	EPDS	BSSR-RI	Physical well-being after delivery	Predictability	Severity of labor
EPDS		–0.278**	–0.398**	0.121	0.206**
BSSR-RI			0.066	-0.399**	–0.519**
Physical well-being (after delivery)				0.041	-0.064
Predictability					0.419**

Note. Rho, ** p <0.01, * p <0.05; the statistically significant correlations are **in bold**. EPDS = Edinburgh Postnatal Depression Scale.BSS-RI = Birth Satisfaction Scale Revised Indicator.

No statistically significant relationship was found between the mode of delivery (vaginal (VB), emergency caesarean (ECS), or elective caesarean section (ELCS)), and the level of PPD. Neither did these groups differ in the quality of physical well-being after childbirth.

Satisfaction with childbirth was significantly lower in women who had emergency caesarean sections than in those who gave birth vaginally (H = 8.17; p = 0.017). VB and ELCS women did not significantly differ in their levels of childbirth satisfaction. ECS mothers perceived their delivery as being more difficult than VB and ELCS mothers did (H = 19.22; p < 0.001, and H = 17.26; p < 0.001, respectively). Similarly, the ECS mothers’ expectations about the childbirth tended to differ more often from the reality than those of VB mothers (H = 8.42; p = 0.015) and ELCS mothers (H = 8.21, p = 0.003).

In our study, 36.1% (n = 69) of the participants gave birth unaccompanied, while 69.3% (n = 121) had individual support (*Table 1*). Women who were accompanied by a midwife and a partner during childbirth showed the highest average level of childbirth satisfaction (*Table 1*). Significantly higher birth satisfaction scores occurred in the group with doula/midwife and partner support, compared to unaccompanied birth (H = -3.32; p = 0.001).

Childbirth satisfaction and well-being after childbirth did not significantly differ based on the country in which the delivery took place. However, there were significant country differences in the support given at the time of delivery, so that in Russia, women gave birth to a child without the support of her partner, a personal midwife or a doula more often than in Europe and Israel (Χ^2^ = 34.844; p < 0.001).

Multiple linear regression analysis showed that levels of birth satisfaction, physical well-being after delivery, perceived severity of the birth experience, and the predictability of labor process contributed significantly to the regression model F (7, 183) = 8.80; p< 0.001, and accounted for 26% of the variance in PPD. Significant predictors of PPD were physical well-being after delivery (B = -2.504; p < 0.001) and birth satisfaction (B = -0.481; p = 0.007). Controlled for maternal age, gestational age at birth, and the number of children the model explained 25% of variance in PPD (*Table 3*).

**Table 3:** Multiple linear regression results examining associations with PPD.

	B	SE	95% CI	p
Model 1				
Birth satisfaction	-.498	.171	-.84:-.16	.004
Physical well-being in 2 months after delivery	-2.542	.395	-3.3:-1.7	.000
Model 2 (adjusted for maternal age, gestational age at birth, and parity as covariates)				
Birth satisfaction	-.481	.177	-.83:-.13	.007
Physical well-being in 2 months after delivery	-2.504	.404	-3.3:-1.7	.000

*Note. B = Unstandardized B. SE = Standard Error. 95% CI = 95% Confidence Interval. p = p-value.*

## Discussion

To our knowledge, this is the first study to examine the association between PPD and childbirth experience in Russian women. Our results show that lower birth satisfaction and physical well-being two months after delivery were associated with higher levels of PPD.These findings corroborate previous studies indicating psychologically and physically traumatic childbirth experiences as a serious risk factor for depression after childbirth (Yildiz, Ayers, & Phillips, 2017). It is further in line with the evidence that acute postpartum pain and persistent pain after delivery are associated with increased risk of PPD (Eisenach et al., 2008).

Our study showed that the mode of delivery (vaginal, emergency caesarean, or elective caesarean section) did not directly correlate with the risk of developing PPD. However, an emergency cesarean section seems to be harder for a woman, as this delivery mode was associated with lower childbirth satisfaction and the realities that fell short of the expectations. Subjectively, such childbirth is perceived as being more difficult.

There are contradictory findings in this regard, with some studies reporting elevated risk of PPD following emergency cesarean section (Yang, Shen, Ping, Wang, & Chien, 2011), while others find no such association (Eckerdal et al., 2018), as is the case in our study. This may be because the important risk factor for postpartum depression is not a mode of delivery per se, but the emotions the woman is experiencing – a fear of an unexpected outcome, a fear for her baby’s health, a feeling of guilt, etc. For example, mothers who had a strong antepartum preference for vaginal delivery, and then delivered by cesarean, may be at increased risk for depression in the early postpartum period (Houston et al., 2015). Importantly, this involves the quality of the support the woman gets from relatives and specialists (Noyman-Veksler, Herishanu-Gilutz, Kofman, Holchberg, & Shahar, 2015).

Our study’s findings indicate that the level of childbirth satisfaction in Russia does not significantly differ from that in European countries and Israel. Specific to childbirth in Russia were lower rates of partner and doula support during the childbirth (especially in the provinces of Russia). This is the legacy of the Soviet system of obstetrics (Temkina, 2014). The presence of a partner or other family member during labor was only allowed in state hospitals beginning in 2012; nowadays there is a growing trend for an increased number of deliveries with the partner present (Novkunskaya, 2020).

Current Federal legislation in Russia does not establish woman’s right to have more than one companion during labor. However, our study shows that childbirth with a doula or individual midwife and a partner’s support is associated with higher levels of birth satisfaction and physical well-being after delivery. These results are consistent with the research data on the positive impact of doula assistance on the psychological well-being of women in childbirth (McLeish & Redshaw, 2019). Thus, a doula may be a mediating specialist who helps the couple to go through the challenging birth experience (Lanning & Klaman, 2019). The results of our research emphasize the importance of the individual professional support during labor and its possible application for the prevention of PPD in Russia.

## Conclusion

Our study showed that birth satisfaction and physical well-being two months after delivery were inversely correlated with PPD. Perceived severity of labor and worse well-being after delivery were associated with lower birth satisfaction. The presence of a partner and a personal midwife or doula at birth was associated with higher birth satisfaction, which indicates the importance of the individual professional support during labor as a possible avenue for PPD prevention.

## Limitations

We used self-report methods to assess the levels of depression. Clinical interviews would supplement the results and make them more valid. A more detailed questionnaire about the woman’s birth experience or a qualitative study would enrich the results about the association between birth satisfaction and perinatal affective disorders. Further research with the larger group with doula or midwife support is needed.
